# Supplementary material for: Pain reduction by inducing sensory-motor adaptation in Complex Regional Pain Syndrome (CRPS PRISMA): protocol for a double-blind randomized controlled trial
Source: BMC Neurol. 2020 Feb 19;20:62. doi: 10.1186/s12883-020-1604-z (PMC7031894; doi:10.1186/s12883-020-1604-z)
Supplement: Supplementary file 1 — Additional file 1: Table S1. lists all items from the World Health Organisation Trial Registration Data Set. [file 12883_2020_1604_MOESM1_ESM.docx]

Table S1 *World Health Organization Trial Registration Data Set*

| Data category | Information |
| --- | --- |
| Primary registry and trial identifying number | International Standard Randomised Controlled Trial Number ISRCTN46828292 |
| Date of registration in primary registry | 27/03/2017 |
| Secondary identifying numbers | Oxford A REC 12/sc/0667, University of Bath Psychology Ethics Committee 16-333 |
| Source(s) of monetary or material support | Reflex Sympathetic Dystrophy Syndrome Association (USA) |
| Primary sponsor | University of Bath (UK) |
| Secondary sponsor(s) | NA |
| Contact for public queries | Ms Monika Halicka ([m.halicka@bath.ac.uk](mailto:m.halicka@bath.ac.uk)), Dr Janet Bultitude ([j.bultitude@bath.ac.uk](mailto:j.bultitude@bath.ac.uk)) |
| Contact for scientific queries | Ms Monika Halicka ([m.halicka@bath.ac.uk](mailto:m.halicka@bath.ac.uk)), Dr Janet Bultitude ([j.bultitude@bath.ac.uk](mailto:j.bultitude@bath.ac.uk)) |
| Public title | Treatment of complex regional pain syndrome (CRPS) with sensory-motor adaptation |
| Scientific title | Pain Reduction by Inducing Sensory-Motor Adaptation in Complex Regional Pain Syndrome (CRPS PRISMA): Protocol for a Double-blind Randomized Controlled Trial |
| Countries of recruitment | United Kingdom |
| Health condition(s) or problem(s) studied | Complex Regional Pain Syndrome |
| Intervention(s) | Active comparator: Prism Adaptation Treatment (two weeks of twice-daily sensory-motor training using 35-diopter Fresnel lenses that induce visual shift away from the CRPS-affected side) |
|  | Placebo comparator: Sham Prism Adaptation (Sham Treatment) (the same procedure using neutral lenses that do not induce visual shift) |
| Key inclusion and exclusion criteria | Inclusion criteria (Participants with CRPS): male/female; age 18-80; CRPS type I primarily affecting one upper limb, meeting Budapest diagnostic research criteria , for >3 months; current pain intensity min. 2/10 |
|  | Exclusion criteria (Participants with CRPS): insufficient English language ability; legally blind; CRPS affecting both sides of the body; CRPS II (confirmed nerve damage); physical limitation preventing execution of Prism Adaptation / sham treatment; severe psychiatric comorbidity |
|  | Inclusion criteria (Healthy control participants): male/female; age 18-80; neurologically healthy; no current or chronic pain |
|  | Exclusion criteria (Healthy control participants): insufficient English language ability; legally blind; physical disability or injury limiting normal mobility; history of neurological or severe psychiatric illness |
| Study type | Interventional |
|  | Allocation: Randomized (with stratification to minimise baseline group differences)  Blinding: Double-blind (Participants with CRPS, outcomes assessor)  Assignment: Parallel |
|  | Primary purpose: treatment |
| Date of first enrolment | 19/06/2017 |
| Target sample size | 42 Participants with CRPS, 21 Healthy control participants |
| Recruitment status | Recruiting |
| Primary outcome(s) | Current self-reported pain intensity on a 0 (no pain) to 10 (pain as bad as you can imagine) Numerical Rating Scale  CRPS severity score based on 16-points scoring system by Harden et al. (*PAIN,* 2017)  Time points: Immediately before the commencement of treatment (week 4) vs. immediately after the end of the treatment period (week 7) |
| Key secondary outcomes | Self-report questionnaires about pain, physical and emotional functioning, body representation, expectations about treatment, and impressions of treatment outcome in weeks 1, 4, 7, 11, 19, and 31 (Brief Pain Inventory – short form, Pain Detect Questionnaire, Bath CRPS Body Perception Disturbance Scale, Tampa Scale for Kinesiophobia, Profile of Mood States); week 1 (Edinburgh Handedness Inventory, Revised Life Orientation Test, Patient-Centred Outcomes Questionnaire); and weeks 7, 11, 19, and 31 (Patient Global Impression of Change)  Self-reported daily ratings of average pain intensity, range of movement, and the extent to which the CRPS symptoms interfere with daily life (weeks 1 to 11)  Clinical assessments of CRPS signs and symptoms and sensory, motor, and autonomic function in weeks 1, 4, 7, and 11 (CRPS severity score, limb temperature asymmetry, oedema, grip strength, delta finger-to-palm distance, mechanical detection threshold, mechanical pain threshold, mechanical allodynia, two-point discrimination threshold)  Computer-based tests of visuospatial attention (Temporal Order Judgement, Landmark, and Greyscales tasks), cognitive representation of space (Mental Number Line Bisection task), spatially-defined motor function (Directional Hypokinesia task), and body representation (Hand Laterality Recognition task) in weeks 1, 4, 7, and 11 |
