# Supplementary material for: Pain reduction by inducing sensory-motor adaptation in Complex Regional Pain Syndrome (CRPS PRISMA): protocol for a double-blind randomized controlled trial
Source: BMC Neurol. 2020 Feb 19;20:62. doi: 10.1186/s12883-020-1604-z (PMC7031894; doi:10.1186/s12883-020-1604-z)
Supplement: Supplementary file 2 — Additional file 2. Supplementary text describing the procedure and results of a pilot study to select the stimulus set for the Hand Laterality Recognition task. [file 12883_2020_1604_MOESM2_ESM.docx]

*Supplementary text describing the procedure and results of a pilot study to select the stimulus set for the Hand Laterality Recognition task*

To develop a stimulus set for the Hand Laterality Recognition task, we photographed a gender-neutral right hand in 36 different postures and mirror-reversed the images to create equivalent pictures of a left hand. In a pilot study, these images were presented at four different orientations (0°, 90°, 180° and 270°), each in left and right visual field (giving a total of 576 trials). In each trail, a black 0.1° fixation cross on a white background was on constant display. After 1000ms a colour image of a hand (12° x 12°) was randomly presented 8° to the left or to the right of the fixation cross (i.e., in the left or the right visual field) for 200ms. The pilot participants (*N =* 22 healthy adults; 11 females; mean age = 27.91; *SD* = 8.43) were required to indicate whether the image represented the right or the left hand by pressing “up” or “down” keys, respectively, using the index and middle fingers of their dominant hand. The next trial started after 3000ms from the stimulus onset or once the response was given, whichever came first. Participants were instructed to be as fast and as accurate in their responses as possible. Following a practice session with longer stimulus presentation times (2000ms), participants completed the task in three blocks, allowing a break after every 196 trials. Fifty images (25 of each hand in the same posture and orientation) were selected from the pilot image bank for the current study based on sufficient accuracy obtained in the pilot study: at least 72% accuracy averaged across both hemifields and less than 15% difference in accuracy between left and right hemifield. These requirements were set to ensure that the stimuli properties were such that a healthy participant is able to determine the laterality above the chance level. Example stimuli are presented in Figure 6 in the manuscript.
